# Convergent mechanisms of epithelial cell structure manipulation by intestinal pathogens

**DOI:** 10.1371/journal.ppat.1013367

**Published:** 2025-07-31

**Authors:** Elena Rodrigues, N. Bishara Marzook, Mitchell A. Pallett, Adam Sateriale

**Affiliations:** Cryptosporidiosis Laboratory, The Francis Crick Institute, London, United Kingdom; Joan and Sanford I Weill Medical College of Cornell University, UNITED STATES OF AMERICA

## Abstract

The epithelial layer that lines the digestive system serves as the primary barrier to infection by intestinal pathogens. While this layer has evolved complex molecular mechanisms to identify and respond to infection, pathogens have also evolved equally complex mechanisms to subvert this response and remodel the epithelium to their benefit. The structure of the intestinal epithelial cell is a common target of this remodeling effort. This review focuses specifically on the phenotypes and mechanisms of epithelial cell structure manipulation that have convergently evolved in human intestinal pathogens.

## Introduction

**Fig 1:**
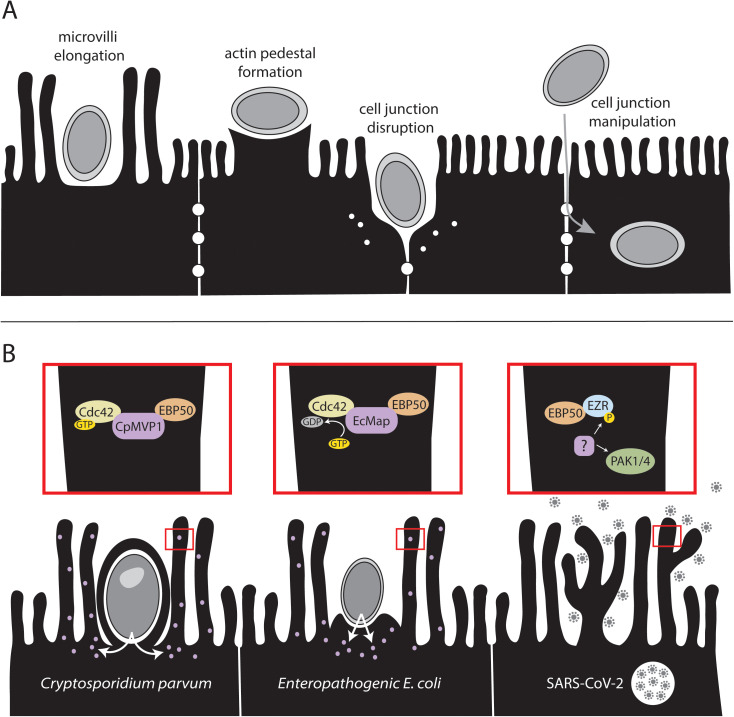
Convergent strategies and mechanisms of epithelial cell structure manipulation by intestinal pathogens. **A)** Overview of mechanisms included within this review. **B)** Overview of microvilli elongation mechanisms of the *Cryptosporidium* parasite (left), Enteropathogenic *Escherichia coli* (center), and SARS-CoV-2 (right).

One of the principal ways that pathogens modulate their infected host is through the manipulation and rearrangement of the cytoskeleton. This modulation may be a direct attempt to enhance pathogen survival and dissemination, or rather an off-target effect of some other virulence mechanism. There is a wealth of literature concerning these cytoskeletal manipulations by both intracellular and extracellular pathogens within the intestine. Here, we focus on phenotypes and mechanisms that appear to be convergent across kingdoms of life; instances where prokaryotic and eukaryotic pathogens have evolved to cause similar alterations within the epithelium of the host intestine. These include: microvilli elongation, actin pedestal formation, and the disruption and manipulation of epithelial cell junctions (Fig 1).

## Elongation of microvilli

Microvilli are thin plasma membrane protrusions, internally reinforced by F-actin and found on the surface of a variety of different cell types [1]. On intestinal epithelial cells, microvilli display apical polarity, facing into the lumen. They provide the primary surface for nutrient absorption and osmoregulation in the gastrointestinal tract [2]. Enteric pathogens first interact with the host at this epithelial brush border, so it is perhaps not surprising that microvilli are targeted for manipulation. Most commonly, this is seen in the destruction of host microvilli. During infection with the enteric bacteria *Vibrio parahaemolyticus,* for example, microvilli loss appears to occur by the process of membrane vesiculation [3]. In contrast, *Salmonella* deploys two distinct mechanisms which can induce microvilli effacement, firstly through villin-mediated F-actin depolymerization and secondly, through the depletion of cytoplasmic G-actin by the redirection to membrane ruffle formation. Both of these phenotypes are mediated through the interaction of virulence factor SopE with the small GTP-binding protein (GTPase) Rac1, a protein known to be involved in cytoskeletal reorganization [4].

**Fig 2:**
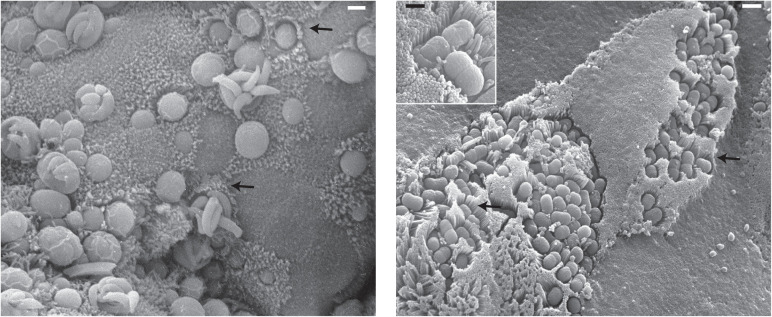
Microvilli elongation in the intestine by the eukaryotic pathogen *Cryptosporidium* and prokaryotic pathogen *Escherichia coli.* Left panel—scanning electron microscopy of murine small intestine infected with *Cryptosporidium parvum* (scale bar 2 μm) (image courtesy of David Ferguson, Oxford University, copyright retained). Right panel—scanning electron microscopy of Enteropathogenic *E. coli* (O127:H6) in the murine small intestine (scale bar 0.5 and 0.2 μm (inset)) (reprinted with permission from the publisher [116]). Black arrows highlight areas with pronounced microvilli elongation.

Enteropathogenic *Escherichia coli* (EPEC) also induces microvilli effacement during sustained infection [5,6]. However, during early infection, the host microvilli surrounding the bacteria are first seen to increase in number, elongate, and stretch toward the pathogen [7]. This well-characterized elongation dramatically increases the cell surface area of infected intestinal epithelial cells, while causing a negligible difference to cytosolic volume. This could allow for increased nutrient uptake into the host cell, better facilitating pathogen survival and preventing host cell death due to nutrient depletion. *E. coli* is not the only enteric pathogen able to manipulate host cell microvilli. Elongation of host intestinal cell microvilli during infection with the eukaryotic intracellular parasite *Cryptosporidium* has also long been seen as a phenotypic hallmark of infection [8–10] (Fig 2). While the mechanism of elongation induced by secreted virulence factors of EPEC has been well-studied, the mechanism by which *Cryptosporidium* is able to drive microvilli elongation has only begun to be explored.

EPEC injects bacterial effector proteins into the infected host cell through specialized protein transport machinery known as the type III secretion system (T3SS). One such effector is the virulence factor known as Mitochondrial-Associated Protein (Map) [11]. Upon entry into the host cell, Map binds to the human apical scaffold protein ezrin-radixin-moesin-binding phosphoprotein (EBP50) via a C-terminal TRL motif [7]. EBP50 is a scaffold protein with apical polarity, and therefore, this interaction is thought to help stabilize and sequester Map at the apical cell membrane within the epithelial cell. At the apical membrane, through a separate guanine nucleotide exchange factor (GEF) domain, Map has been demonstrated to bind and activate the Rho GTPase Cdc42 [12,13]. Cdc42 is a potent activator of neural Wiskott-Aldrich syndrome protein (N-WASP) which further activates the Arp2/3 complex, a protein complex that regulates the actin cytoskeleton within eukaryotic cells [14]. This signaling cascade leads to increased nucleation of F-actin present in the host microvilli and subsequently elongation [13].

While a *Cryptosporidium* homolog of Map does not exist, an exported virulence factor of the parasite has recently been discovered that appears to have convergently evolved to modulate microvilli length through a very similar mechanism [15]. MVP1 is a highly transcribed *Cryptosporidium* protein that is exported into the infected host epithelial cell, where it localizes to the apical membrane. Here, through a C-terminal TRL domain, MVP1 binds to host EBP50 in a similar fashion to Map. However, unlike Map, MVP1 does not contain a GEF domain that directly activates Cdc42. However, MVP1 appears to contain a Cdc42- and Rac-interactive binding (CRIB) domain, which is known to preferentially bind the active form of Cdc42. Without MVP1, microvilli elongation no longer occurs within *Cryptosporidium*-infected epithelial cells. Further, it was found that the deletion of the CRIB domain of MVP1 alone was sufficient to completely ablate the microvilli elongation phenotype [15]. The discovery of this mechanism in *Cryptosporidium* highlights an interesting occurrence where eukaryotic and prokaryotic virulence factors have convergently evolved to modulate host cell structure through the same host signaling pathways.

Elements of this convergence may also be shared by other pathogens. For example, under physiological conditions, invading *Salmonella* appear to cause elongation of the surrounding microvilli. The *Salmonella* effector SipA has been shown to bind directly to actin and, working with the effector SipC, stimulates actin nucleation. It has been proposed that this function of SipA could be responsible for the microvilli elongation phenotype observed during infection [16]. It has also recently been shown that SARS-CoV-2 drives an elongation of microvilli from infected nasal epithelial cells [17]. Curiously, these elongated microvilli appear to be branched as well. EBP50 and phosphorylated ezrin were found to be enriched within infected cells at the epithelial brush border. Treatment with microvilli formation inhibitors, and PAK1 and PAK4 inhibitors, lead to decreased phospho-ezrin recruitment and decreased infection within nasal epithelial cultures, yet the elongation mechanism is still unclear. It is suggested that the purpose of microvilli elongation, in the context of SARS-CoV-2 infection, is to aid in viral egress. The purpose of microvilli elongation in the intestine, for either *Salmonella*, EPEC, or *Cryptosporidium*, is still yet to be determined.

## Actin pedestal formation

Pathogenic *E. coli* and *Cryptosporidium* share another common modulation of the host cytoskeleton—formation of actin pedestals. These actin-dense structures protrude from infected epithelial cells and sit directly underneath the infecting pathogen. While the mechanism driving their formation is well described in *E. coli*, their purpose is still debated [18,19]. The subgroup of pathogenic *E. coli* includes EPEC and Enterohemorrhagic *E. coli* (EHEC) [20]. While EPEC is primarily an infection of the small intestine, EHEC predominantly infects the large intestine [21]. Although EPEC and EHEC have different mechanisms of driving actin pedestal formation, both start with the insertion of Tir via the T3SS [22,23]. During EHEC infection, Tir is thought to recruit another virulence factor, EspFu, through interactions with host proteins IRTKS and IRSp53 [24]. EspFu then binds the autoinhibitory region of WASP, activating N-WASP [25]. During EPEC infection, Tir is thought to recruit the host protein Nck, which can either activate N-WASP directly or indirectly [26,27]. Activated N-WASP then recruits Arp2/3 to drive actin polymerization and the formation of the actin pedestal [28]. A number of other virulence factors have been demonstrated to participate in this process, notably Map, EspH, and NleL, yet their roles are not as clearly defined as central to the consensus mechanism [12,29,30].

Much less pronounced than the pathogenic *E. coli* actin pedestal, the *Cryptosporidium* parasite produces a similar phenotype when it infects epithelial cells of the small intestine [31,32]. *Cryptosporidium* is an intracellular parasite that invades the apical side of epithelial cells in the gut. It has been shown that the parasite engages the host actin cytoskeleton throughout this invasion process, driving the formation of a protective vacuole of host membrane around itself [33–35]. The parasite then maintains this actin-dense band or pedestal underneath the ‘parasitophorous vacuole’ while it replicates. Although the formation of an actin pedestal by *Cryptosporidium* was established over 25 years ago, the mechanism by which the pedestal is formed and maintained is still unknown. What is known suggests that the mechanism might converge on some of the same host pathways as pathogenic *E. coli*. In an intestinal epithelial cell monolayer model of infection, N-WASP, VASP, and Arp2/3 have all been localized to the site of infection [33]. However, there are no readily identifiable *Cryptosporidium* homologs to the *E. coli* Tir or EspFu proteins.

While the ‘how’ of actin pedestal formation in pathogenic *E. coli* is well studied, the ‘why’ is still unclear. It is well supported, through *in vivo* models of *E. coli* infection such as the tractable murine model *Citrobacter rodentium*, that without Tir there is a decrease in pathogen fitness, as Tir mutants result in decreased epithelial colonization within infected hosts [36,37]. It has been proposed that the actin pedestal of *E. coli* serves to (1) anchor the bacteria to the epithelial cell, (2) aid in the delivery of virulence factors, (3) enable for actin-based motility, and (4) protect from phagocytosis [19,38–40]. The *Cryptosporidium* parasite may form an actin pedestal for similar motives, although there is no evidence to support actin-based motility of the parasite once it is within the host cell. It is also tempting to speculate that the actin pedestal and microvilli elongation are intertwined, as *Cryptosporidium* and EPEC share these phenotypes. However, in *Cryptosporidium* and EPEC formation of the actin pedestal and microvilli elongation appear to be primarily driven by separate virulence factors.

## Disruption of cell junctions

Intercellular junctions, including the adherens junctions (AJ) and tight junctions (TJ) are crucial for maintaining intestinal barrier integrity, enterocyte cell polarity, and a vital first barrier to limiting infection. It is no surprise that many enteric pathogens have evolved diverse mechanisms to target and disrupt AJs and TJs and epithelial barrier integrity [41,42]. Disruption of intercellular junctions is a key feature of enteric pathogen infection and critical for virulence resulting in intestinal permeability and paracellular translocation of pathogens. The nature of the interaction between the host intercellular junctions and pathogens is complex and diverse, and the mechanism of disruption can be either direct or indirect.

A clear example of direct targeting is the highly conversed virulence factor family of high-temperature requirement A (HtrA) serine proteases. HtrAs through a trypsin-like protease domain are integral for survival in response to heat and oxidative stress, degrading and refolding misfolded proteins [43,44]. The most well-characterized HtrA member for intercellular junction disruption is the *Campylobacter jejuni* HtrA. Essential for virulence, surface-bound *C. jejuni* HtrA [45] directly degrades adherens and tight junction proteins occludin, claudin-8, and E-cadherin [45–47]. Many major Gram-negative gastrointestinal pathogens encode homologs to HtrA (DegP/Q/S) including EPEC, *Salmonella enterica* Typhimurium (STm), *Shigella flexneri*, *Yersinia enterocolitica*, *Vibrio cholerae* and *Acinetobacter baumannii* as well as the Gram-positive *Bacillus subtilis* (YkdA) [48–52] and the gastric pathogen *Helicobacter pylori* [53]. Although not conserved through the expression of homologous proteins, many enteric pathogens deliver alternative proteases to accomplish the same function. *Entamoeba histolytica* delivers EhCPADH/EHCP112 [54], *Aeromonas hydrophilia* secretes Ssp1 [55], *Giardia intestinalis* secretes a cysteine protease [56], and *Vibrio cholerae* secretes a hemagglutinin protease [57].

Disruption of cellular junctions can also be indirect, as the host cytoskeleton is intimately connected to the apical junctions. Modulation of the cytoskeleton is key to enteric pathogen infection and is essential for attachment, invasion, and dissemination [58]. Ultimately, these pathways can lead to AJ/TJ protein mis-localization and cell junction destabilization [59,60]. Two examples of conserved mechanisms that enteric pathogens use to modulate the host cytoskeleton are (1) effectors that directly modulate actin through cross-linking and/or ADP-ribosylation [61], and (2) effectors that subvert or mimic small GTPases to modulate intracellular signaling and cytoskeletal dynamics [62].

The largest family of virulence factors that target actin are known as mARTs or mono-ADP-ribosyl-transferases. These virulence factors exist as either binary toxins performing both pore formation and translocation of the enzymatic domain into the host cytoplasm, or as single effector domains that are translocated by specialized bacterial secretion systems. Such examples of pathogens harboring these multi-subunit toxins include *Clostridium perfringens* (Iota), *Clostridium botulinum* (C2), *Clostridium spiroforme* (Sa) and *Clostridium difficile* (CDTa). Alternatively, STm (SpvB), *A. hydrophilia* (AexT), and *V. cholerae* (VrgG-1) deliver their effectors through targeted secretion [63–65]. The ADP-ribosyltransferase activity of these toxins directly links ADP-ribose in the presence of NAD+ to arginine 177 of host actin leading to sequestration of G-actin and subsequent collapse of F-actin polymers. Investigations for *Clostridium* mARTs and SpvB of STm have shown that these mARTs induce redistribution or downregulation of claudin-1/4, occludin, and E-cadherin [66,67], cell rounding, and eventual cell death [68–70]. While the potential role of all mARTs in apical junction disruption remains to be investigated it is worth emphasizing that SpvB is essential for virulence and bacterial dissemination in mouse models of STm infection, highlighting the importance of actin ribosylation and TJ disruption in bacterial infection [66,71].

Another well-known example of virulence factors that modulate cellular junctions are the WxxxE domain-containing effectors [62]. The EPEC effector Map, as previously noted, contains a WxxxE domain and is a GEF mimic which activates Cdc42 [12,62,72]. EPEC is known to decrease transepithelial electrical resistance, and this can be rescued by the deletion of MAP [73]. Map, along with the effector EspF, is required for intestinal barrier dysfunction in the mouse model of *Citrobacter rodentium* infection [74]. Similarly, STm translocates WxxxE domain-containing effectors SopE and SopE2, which are also GEF mimics, which activate Rac1 and Cdc42 [75]. Along with the effectors SopB and SopE/E2, STm drives ZO-1 and occludin mis-localization and epithelial barrier damage [4,76]. The current model proposes that Rac1-dependent depletion of local G-actin pools leads to loss of TJ integrity [4]. This is supported by the fact that small GTPases, such as Rac1 and Cdc42, are essential for the formation and maintenance of TJs in epithelial cells [77,78]. As previously noted, the eukaryotic pathogen *Cryptosporidium* expresses a virulence factor, MVP1, that binds activated Cdc42. Whether MVP1 plays a role in cell junction integrity remains to be investigated; however, *Cryptosporidium* is well-known to disrupt cellular junctions within the intestine [79–81].

## Manipulation of cell junctions

Apical junctional complexes have roles beyond this physical barrier, including the establishment of cell polarity, regulating the actin cytoskeleton, and relaying downstream signaling processes affecting cellular proliferation, differentiation, stress response, and cell survival (for reviews see [82,83]). Many enteric pathogens co-opt or manipulate these downstream roles, rather than target them directly for disruption.

The most direct example of convergent co-option of a TJ protein by pathogens is the eponymous coxsackievirus and adenovirus receptor (CAR), which is an integral part of epithelial TJs co-localizing with occludin and ZO-1 [84,85]. CAR is a definitive receptor for the entry of both group B coxsackieviruses which are part of the enterovirus family, and most adenoviruses causing gastrointestinal disease [86,87]. Further work showed that while coxsackie B virus does not significantly disrupt the TJ barrier, it causes internalization of TJ protein occludin during virus entry [88,89]. More recently, the rhesus enteric calicivirus, a pathogen used to model human norovirus, was shown to also require CAR for cell entry [90], which is intriguing as the receptor for human norovirus entry remains unknown.

Eukaryotic pathogens such as *Toxoplasma gondii*, which is transmitted by the oral route and where the gut is the first port of entry, require occludin for successful transmigration between epithelial cells in vitro; however, invasion of cells themselves was less impacted by occludin loss [91]. SARS CoV-2 primarily causes lung infections but can also infect enterocytes [92]. Occludin is required for SARS-CoV-2 internalization and replication in lung and gut epithelial cells *in vitro*, mediated by its Spike protein [93]. Rotavirus, the leading cause of diarrheal disease and death in infants, also requires occludin for host cell entry, along with ZO-1 and JAM-A in a strain-dependent manner as their siRNA-mediated loss reduces viral infectivity [94]. This TJ interaction is mediated by viral capsid protein VP8 [95], and results in RhoA-ROCK activation in a protein kinase A (PKA)-dependent manner [96,97]. Occludin is therefore a host TJ protein co-opted by varied enteric pathogens across multiple kingdoms of life.

Another TJ-associated signaling pathway manipulated by intestinal pathogens is that of the myosin light chain kinase (MLCK), which controls TJ permeability, proper distribution of ZO-1 and occludin, and development of diarrhea [98–100]. Intestinal pathogens express effectors that activate MLCK, causing increased phosphorylation of myosin light chain and eventual loss of barrier integrity. For example, *Listeria monocytogenes* adhesion protein LAP activates MLCK *in vitro* and in mice, and the loss of epithelial barrier integrity is dependent on MLCK, since *Mlck*^−/−^ mice are protected from barrier loss and systemic *Listeria* infection [101]. Additionally, infection of epithelial cells with EPEC has been known to increase MLC phosphorylation, leading to perturbation of the intestinal epithelial barrier [102]. Similarly, gastric pathogen *H. pylori* activates MLCK, leading to loss of TJ barrier function via its protein UreB [103,104]. The eukaryotic pathogen *Giardia intestinalis* also alters barrier integrity and causes redistribution of F-actin and ZO-1 in an MLCK-dependent manner [105]; however, the precise effector responsible is unknown.

Atypical protein kinase C (aPKC) maintains strict epithelial polarity through its apical junctional complex and kinase functions [106,107] and is targeted by pathogens in different ways. EPEC effector EspF has been known to disrupt barrier function, in part by causing internalization of aPKC-associated proteins at apical junctions, but recent work has highlighted that recruitment of aPKC to EPEC actin pedestals is the first step in this process, triggering a cascade of mis-localization of various junctional proteins to the cytoplasm, which eventually leads to loss of apico-basal polarity and barrier integrity [108,109]. Conversely, *H. pylori* effector CagA, which disrupts TJ by recruiting ZO-1 [110], does so by recruiting aPKC and other members of its polarity complex, thus preventing the aPKC-mediated phosphorylation of PAR1, which usually ensures its proper basal localization and the maintenance of cell polarity via a feedback mechanism [111,112]. In this way, pathogenic effectors cause the loss of proper cell polarity and TJ-mediated cytoskeletal integrity by manipulating upstream signaling pathways.

Whether the pathogen directly disrupts tight junctions through an effector or does so indirectly by manipulating another cellular process, the ultimate consequence is a weakening of the epithelial barrier. Why then have so many enteric pathogens evolved to erode the epithelial barrier? Dissemination is a likely culprit. Disrupted epithelial barriers allow for systemic pathogens to escape the confines of the intestine and invade other organs. Disrupted barriers also lead to inflammation and diarrhea, increasing the likelihood of pathogen transmission, but also jeopardizing the health of the current host. There is evidence that some pathogens have evolved effectors with opposing functions to hedge their bets. *Salmonella’s* AvrA protein appears to stabilize TJs, the loss of which enhances gut permeability [113], and the *Entamoeba histolytica* secreted protein EhADH stabilizes TJ proteins even though it has a contrasting function when in complex with the cysteine protease EhCP112 [114,115]. Likely, these effectors are only a small subset of effectors that evolved to strike a balance between virulence and infection persistence.

With so many convergent mechanisms to manipulate the structure of epithelial cells, one has to wonder how the complex nature of the intestinal microbiota influences their evolution. Every mechanism in this review has been described in controlled systems with a single pathogen. Yet co-infection is common in vertebrates and humans living within endemic areas of intestinal disease. Did certain mechanisms converge in response to competition? Or were these mechanisms simply the most effective path of host cell manipulation? Scaling up to more physiological and complex systems may help shed light on some of these salient questions.
